# Frontier progress and translational challenges of pluripotent differentiation of stem cells

**DOI:** 10.3389/fgene.2025.1583391

**Published:** 2025-04-28

**Authors:** Zhengbing Su, Hui Dong, Xiang Fang, Wenli Zhang, Hong Duan

**Affiliations:** Department of Orthopedic Surgery, West China Hospital, Sichuan University, Chengdu, China

**Keywords:** pluripotent stem cells, regenerative medicine, stem cell differentiation, disease modeling, stem cell therapy

## Abstract

Stem cell research has significantly transformed regenerative medicine, with pluripotent stem cells (PSCs) serving as the cornerstone for disease modeling, drug screening, and therapeutic applications. Embryonic stem cells (ESCs) exhibit unparalleled self-renewal and tri-lineage differentiation, while induced pluripotent stem cells (iPSCs) bypass ethical constraints through somatic cell reprogramming. Clinical trials highlight the potential of mesenchymal stem cells (MSCs) in osteoarthritis and graft-versus-host disease, which leverage their immunomodulatory and paracrine effects. Despite advancements, challenges persist: iPSCs face epigenetic instability and tumorigenic risks, and adult stem cells struggle with inefficient differentiation. This paper systematically reviews stem cell source classification, differentiation regulatory mechanisms, cutting-edge technologies such as CRISPR/Cas9, and explores field-specific controversies (e.g., epigenetic stability of iPSCs) and future directions (e.g., integration of organoids and biomaterials). By analyzing current progress and challenges, it provides a multidimensional perspective for stem cell research.

## 1 Introduction

The emergence of induced pluripotent stem cells (iPSCs) in 2006 marked a paradigm shift, allowing patients to receive specific treatments without damage to embryos. However, some critical debates still persist, including the following: (1) Source-specific regulation: How do transcriptional networks differ between ESCs, iPSCs, and adult stem cells? (2) Ethical dilemmas: While iPSCs circumvent the use of embryos, chimeric models and germline editing remain contentious. (3) Standardization gaps: Heterogeneity in MSC batches and variability in iPSC differentiation protocols hinder their clinical scalability. This article integrates research findings and focuses on three major directions: the classification and characteristics of stem cells, cutting-edge technologies for differentiation regulation, and bottlenecks in clinical translation. This study aims to provide a systematic analysis for the development of this field.

## 2 Types and sources of stem cells

### 2.1 Embryonic stem cells

Embryonic stem cells (ESCs) are pluripotent cells derived from the inner cell mass of blastocyst-stage embryos obtained from mice or humans. They possess pluripotency and can be infinitely expanded *in vitro* without undergoing replicative senescence or aging (Lorzadeh and Kazemirad, 2018; Zhang et al., 2025). Building upon prolonged *in vitro* culture techniques for human embryos, researchers have systematically characterized the developmental dynamics and molecular signatures of distinct cell lineages. A recent study revealed critical functional roles of FGF, Wnt, and BMP signaling pathways in lineage specification and further elucidated potential mechanisms underlying the aberrant development of aneuploid embryos (Shahbazi et al., 2020; Molè et al., 2021; Ai et al., 2023). However, the application of human embryonic stem cells (hESCs) raises some ethical issues, including the following: 1) the vague definition of the moral status of embryos. Although embryos within 14 days can be used to save patients, their biological life needs to be respected; 2) undesirable practices in informed consent, such as inducing participants to sign documents and concealing intended uses; 3) risks of harm, including patient health issues caused by defects in cloning technology, as well as risks to gamete providers in underground markets; 4) ethical concerns regarding the dignity and safety of chimeric embryos; and 5) challenges in benefit distribution, including result-sharing, interest allocation, and conflicts between embryos and patients—ultimately requiring a balance of interests (Mountford, 2008; Bhartiya et al., 2013; Luk et al., 2017). Therefore, current related research is mainly limited to animal experimentation, and clinical translation still lacks sufficient data to support its safety and efficacy.

### 2.2 Adult stem cells

Adult stem cells (ASCs) are present in various tissues and organs of adults, such as the bone marrow, skin, brain, and intestine. Under normal circumstances, they remain in a quiescent state but are activated to participate in tissue repair in the presence of tissue damage. They include hematopoietic stem cells (HSCs) and mesenchymal stem cells (MSCs), and so on. Although their differentiation potential is limited, they have a relatively high level of safety. In the future, through the integration of single-cell sequencing and organoid technologies, adipose-derived stem cells (ADSCs) are likely to become the core tools for personalized medicine and disease modeling, bridging the gap between safety and functionality in precision medicine.

#### 2.2.1 Hematopoietic stem cells

Hematopoietic stem cells (HSCs) are pluripotent stem cells that possess the abilities of self-renewal, proliferation, and pluripotent differentiation (Laurenti and Göttgens, 2018; Calvanese et al., 2022). Most HSCs remain stationary in the body, which is an important mechanism for maintaining HSC numbers and hematopoietic balance. Additionally, HSCs maintain a stable state to preserve their self-renewal capacity and protect themselves from genetic damage and excessive stress, thus ensuring their long-term survival and normal function (Chen et al., 2021). Under stressful conditions such as tissue damage and inflammation, HSCs can be activated and enter the cell cycle to begin self-renewal and differentiation in response to these emergencies (Dzierzak and Bigas, 2018). Due to their unique hematopoietic reconstitution ability, hematopoietic stem cell transplantation (HSCT) has become an important method for treating various hematological diseases, including malignant tumors of hematopoietic cells in the bone marrow and immunodeficiency diseases (Demirci et al., 2020). However, the insufficient number of donor cells remains the main bottleneck limiting its clinical application (Domingues et al., 2017; Okeke et al., 2021). In the future, with breakthroughs in gene editing technologies (such as CRISPR) and *in vitro* amplification technologies, it is expected that these advancements will reduce the shortage of donor cells, improve transplantation efficiency and safety, and provide more treatment options for patients.

#### 2.2.2 Mesenchymal stem cells

Mesenchymal stem cells (MSCs) are a type of adult stem cell derived from the mesoderm. They primarily reside in the bone marrow and account for 0.001%–0.01% of the total nucleated cell population. MSCs exhibit characteristics such as self-renewal capacity, chemotactic migration properties, and transdifferentiation potential, making them ideal seed cells for tissue engineering (García-Pagán et al., 2012; Drela et al., 2020). Their unique immunomodulatory function modulates the body’s inflammatory response through the expression of immunosuppressive factors. For instance, MSCs can promote angiogenesis and re-epithelialization, regulate immune activity, reduce inflammation, and ultimately facilitate the repair of diabetic foot ulcers (Yu et al., 2023). A randomized, double-blind, placebo-controlled study investigated the efficacy of a single intraarticular (IA) injection of bone marrow-derived mesenchymal stem cells (BM-MSCs) in patients with knee osteoarthritis (OA). The results demonstrated that a single administration of allogeneic BM-MSCs via IA injection significantly alleviated pain at 9 months compared to the control group. Furthermore, quantitative T2 MRI cartilage mapping revealed that IA BM-MSCs inhibited OA progression over a 12-month period (Lee et al., 2024). In studies on graft-versus-host disease (GVHD), human placental mesenchymal stem cells (hPMSCs) were shown to mitigate GVHD-induced liver injury by reducing the proportion of CD8+PD-1+ T cells via the CD73/ADO/Nrf2 signaling pathway (Yan et al., 2025). Although extensive research supports the role of MSCs in liver regeneration and functional recovery, the therapeutic application of MSC transplantation for liver injuries remains contentious (He et al., 2024; Mincheva et al., 2025; Tian et al., 2025). A critical challenge lies in the *in vivo* microenvironment’s propensity to drive aberrant MSC differentiation into myofibroblasts, promoting fibrotic scar formation instead of functional hepatocyte differentiation (Ichim et al., 2018). This uncontrolled differentiation represents the primary obstacle to achieving therapeutic efficacy.

### 2.3 Induced pluripotent stem cells (iPSCs)

iPSCs are pluripotent cells that are artificially generated through genetic reprogramming of mature somatic cells. Their discovery has overcome some ethical limitations associated with embryonic stem cells (ESCs) and has demonstrated unique advantages (Takahashi and Yamanaka, 2006). Similar to ESCs, iPSCs express pluripotency markers, possess unlimited proliferative capacity, and can differentiate into ectodermal, mesodermal, and endodermal cells (Shi et al., 2017; Omole and Fakoya, 2018). Furthermore, iPSCs can be generated through autologous cell reprogramming or customized generation of cells with specific genetic backgrounds, providing opportunities for personalized medicine (Mianné et al., 2018; Stoddard-Bennett and Reijo Pera, 2019). Unlike ESCs, iPSCs circumvent the need to destroy human embryos and offer flexible applications, enabling non-invasive collection from patients with rare diseases. iPSCs have been widely used to model heart diseases, study hereditary arrhythmias, and investigate neurological disorders (including Alzheimer’s disease), liver diseases, and spinal cord injuries. The discovery of iPSCs has significantly advanced regenerative medicine, unlocking new avenues for harnessing pluripotent cells’ potential (Aboul-Soud et al., 2021). Table 1 summarizes the sources, key characteristics, clinical applications, and major challenges of ESCs, HSCs, MSCs, and iPSCs. Careful comparisons show that stem cells from different sources exhibit significant differences in differentiation potential, ethical constraints, and clinical translation potential.

**TABLE 1:** Classification, source, key characteristics, clinical applications and challenges of stem cells.

Stem cell type	Source	Key characteristics	Clinical applications	Challenges
ESCs	Inner cell mass of blastocysts	Pluripotency, unlimited *in vitro* expansion	Disease modeling, drug screening (Thomson et al., 1998)	Ethical controversies, immune rejection (Luk et al., 2017)
HSCs	Bone marrow, peripheral blood, umbilical cord blood, placenta	Self-renewal and multi-lineage differentiation ability, differentiating into blood cells and immune cells	Bone marrow transplantation for the treatment of hematological diseases (Demirci et al., 2020)	Insufficient number of donor cells (Okeke et al., 2021)
MSCs	Bone marrow, adipose tissue, umbilical cord, placenta, peripheral blood	Immunomodulatory function, trans-germ layer differentiation potential	Tissue engineering, anti - inflammatory therapy (García-Pagán et al., 2012)	Microenvironment-induced abnormal differentiation (Seki et al., 2007)
iPSCs	Reprogrammed somatic cells	ESC-like pluripotency, patient-specific	Personalized medicine (Stoddard-Bennett and Reijo Pera, 2019)	Epigenetic instability (Suresh Babu et al., 2023)

## 3 Regulatory mechanisms of stem cell differentiation

### 3.1 Intrinsic regulatory mechanisms

#### 3.1.1 Transcription factor network

Specific transcription factors maintain the self-renewal ability of stem cells or initiate the differentiation program through synergistic effects. For example, inhibition of the transcription regulator NF-kB (nuclear factor kappa B) impedes the differentiation of the mesoderm and neuroectoderm in mouse and human embryonic stem cells (Kaltschmidt et al., 2021). Furthermore, transcription factor CEBPD-mediated Wilms tumor 1-associated protein (WTAP) promotes the stemness, growth, migration and glycolysis of glioblastoma stem cell-like cells (GSCs) and reduces their tumorigenicity *in vivo* (Geng et al., 2025).

#### 3.1.2 Epigenetic modifications

Epigenetic modifications determine the differentiation fate of stem cells by dynamically regulating gene expression. For example, in clustered regularly interspaced short palindromic repeats/CRISPR-associated protein 9 (CRISPR/Cas9)-mediated ING5 knockout mice, the absence of ING5 leads to low fertility and depletion of stem cell pools in multiple tissues, although it does not reduce lifespan or impair wound healing ability (Al Shueili et al., 2025). Both drug inhibition and siRNA-mediated EZH2 knockout can reduce the level of H3K27me3 near the transcription initiation region, thereby stimulating the expression of related bone genes and promoting the osteogenic differentiation of MSCs (Dudakovic et al., 2018). Patient-derived primary glioblastoma stem cell lines, astrocyte cell lines, and primary fibroblast cell lines were treated with epigenetic drugs and found to produce human leukocyte antigen class I-presenting antigens while minimizing the potential side effects of activating unwanted genomic regions (Jang et al., 2024).

#### 3.1.3 Metabolic regulation

The metabolic state of stem cells is closely related to their differentiation ability. For example, MFN2 and the Wnt/β-catenin signaling pathway co-regulate the glycolysis of iPSC-MSCs. The deficiency of MFN2 promotes aerobic glycolysis and osteogenic differentiation (Deng et al., 2022). During the differentiation of stem cells into astrocytes, GLUT1 expression is reduced, with neural progenitor cells showing the lowest expression levels. Truncated GLUT1 did not affect the differentiation of stem cells into astrocytes. However, astrocytes in GLUT1 deficiency syndrome (GLUT1DS) failed to express full-length GLUT1 at the protein level. Furthermore, glucose uptake, lactate production, glycolysis, mitochondrial activity, ATP levels, and extracellular glutamate release were all reduced (Pervaiz et al., 2025). In an animal model of Werner syndrome accompanied by severe osteoporosis, the restoration of NAD^+^ alleviates mitochondrial dysfunction and extends the lifespan of cells (Oshima et al., 2017; Fang et al., 2019).

### 3.2 Extrinsic regulatory mechanisms

#### 3.2.1 Microenvironment (niche) signals

The microenvironment in which stem cells reside regulates their behavior through mechanical forces and biochemical signals. For example, the extracellular matrix (ECM) enhances mitochondrial fusion by upregulating fusion-related protein expression and suppressing associated inhibitory protein activity, thereby promoting osteogenesis. YAP affects glycolysis, glutamine metabolism, and other metabolic processes; regulates stiffness-mediated osteogenic differentiation; and acts as a mechanical sensor integrating ECM mechanical signals with energy metabolism signals. Additionally, glycolysis regulates YAP activity through cytoskeletal tension-mediated nuclear deformation (Na et al., 2024). Non-selective NO inhibitors inhibit methotrexate-induced Wnt/β-catenin target genes and Lgr5+ cell activity but also enhance sFRP-1 expression. Thus, methotrexate partially mediates intestinal stem cell integrity through NO-dependent Wnt/β-catenin signaling and may enhance tolerance to methotrexate-induced injury (Machida et al., 2022).

#### 3.2.2 Cell interactions

Cytokines such as VEGF and FGF secreted by neighboring cells, as well as miRNAs carried by exosomes, can regulate the differentiation of stem cells. Differentiation signals can also be transmitted through intercellular junctions, such as gap junctions, or through adhesion molecules, such as cadherins. For example, a study confirmed that mesenchymal stem cells (MSCs) promote intestinal mucosal repair by regulating fibroblast-epithelial cell interactions. In the *in vitro* co-culture model, fibroblasts treated with MSC-CM (CCD-18Co) promoted intestinal epithelial cell (Caco-2) proliferation by increasing the positive rate of EdU and accelerated wound healing by enhancing cell migration (Huang et al., 2025). Embryonic stem cells (ESCs) co-cultured with neural stem cells (NSCs) express higher levels of the ectodermal markers PAX6 and SOX1 under two co-culture conditions; however, the differentiation efficiency of the conditioned medium (CM) was lower than that of the non-conditioned medium. The results indicate that co-culture with NSCs promotes the differentiation of ESCs into the ectoderm (Kim et al., 2022).

## 4 Cutting-edge technologies and applications

### 4.1 Gene editing technology

The combination of CRISPR-Cas9 technology and induced pluripotent stem cells (iPSCs) provides a new strategy for the treatment of genetic diseases. For example, Mazzarino et al. used iPSCs and CRISPR-Cas9 technology to establish Alzheimer’s disease brain tissue models derived from both the wild-type and homozygous mutant variants of APOE3Ch. They found that the homozygous mutation of APOE3Ch can resist neurodegeneration and delay disease progression (Mazzarino et al., 2023). The Itoh team discovered that CRISPR-Cas9 technology can correct pathogenic mutations in iPSCs specific to recessive dystrophic epidermolysis bullosa (RDEB), achieving residue-free gene editing via the piggyBac transposon system (Itoh et al., 2020).

### 4.2 Organoid construction

**FIGURE 1:**
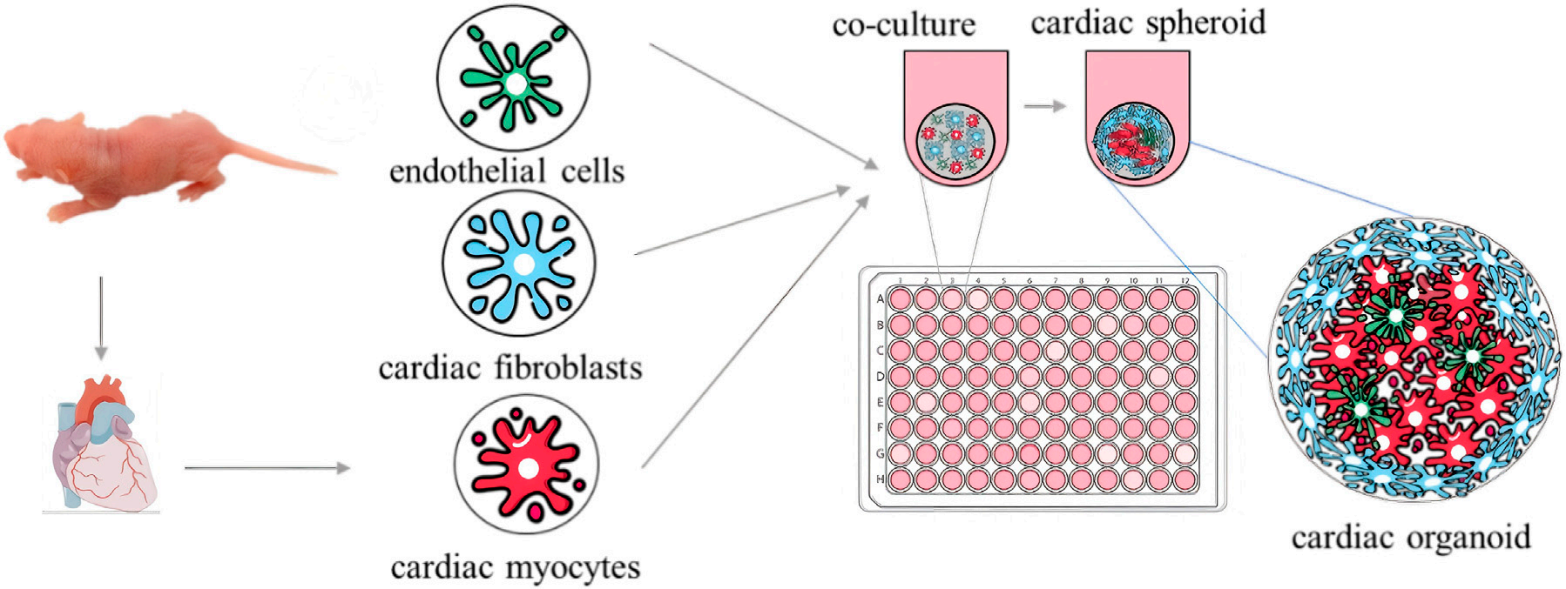
Schematic illustration of 3D co-culture-generated cardiac microspheres.

Breakthroughs have been made in organoid model construction technology, with the research field trending toward the coordinated development of multi-organ systems. In terms of basic model construction, researchers have successively overcome the challenge of simulating multiple organs *in vitro*, such as using 3D retinal pigment epithelial cell models derived from patients (Manian et al., 2021); brain organoids with complex neural activity and optic cup fine structures (Gabriel et al., 2021); patient-derived cervical cancer organoid models (Kutle et al., 2023); 3D pancreatic organoid models (Darrigrand et al., 2024); cardiac organoids characterized by cardiac microsphere formation through 3D co-culture (Figure 1) (Fan et al., 2023); and spontaneous beating scaffold-free human cardiac organoids (Xia et al., 2025). These milestones collectively drive organoid technology from single-organ simulation to the construction of complex physiological environments with multi-system interactions.

### 4.3 3D bioprinting

Using 3D bioprinting promotes the development of personalized medicine through the customization of organs and medical devices. This technology enables precise printing of tissues and organs, such as cardiac tissues, skin, and bones (Omondi et al., 2024), and offers novel approaches for personalized drug manufacturing (Ullah et al., 2023). It has the potential to revolutionize medical device manufacturing by enhancing precision and targeting in tissue engineering and drug delivery (Dedeloudi et al., 2025). The customized bioprinting process not only improves transplantation success rates but also mitigates immune rejection. Additionally, material optimization facilitates achieving a balance between the mechanical properties of the devices and patients’ comfort.

## 5 Clinical transformation bottlenecks and future directions

### 5.1 Technical bottlenecks

The clinical application of stem cells poses significant challenges. The engraftment and survival of transplanted stem cells in myocardial tissue represent the most critical barrier in cell therapy. Preclinical studies (Hou et al., 2005) and clinical trials (Blocklet et al., 2010) demonstrate that cell retention rates fall below 10% within 24 h post-transplantation, with only ∼1% remaining after 4 weeks (Terrovitis et al., 2009). This rapid attrition primarily results from blood flow shear stress, extravasation at injection sites, and cell death. More critically, undifferentiated iPSCs exhibit inherent tumorigenic potential, potentially leading to teratoma formation and genetic aberrations (Sato et al., 2019; Lemmens et al., 2023). Additional risks emerge during reprogramming and *in vitro* culture/expansion, including genomic instability (e.g., chromosomal amplifications and copy-number variations), reactivation of pluripotency transgenes, and mutations in proto-oncogenes/tumor suppressor genes (Sato et al., 2019; Stoddard-Bennett and Pera, 2020; Suresh Babu et al., 2023). These safety concerns collectively constitute major obstacles to stem cell clinical translation.

### 5.2 Future strategies

Artificial intelligence (AI) technology has become an emerging trend. In liver regeneration, AI can automatically segment the liver parenchyma from imaging data such as CT and MRI through upgraded algorithms, achieving “millimeter-level” accurate calculation of the liver volume, which far surpasses traditional manual measurement methods. When combined with imaging tracking technology, it can dynamically generate the regeneration curve after liver resection and identify the inflection point of the regeneration rate, providing an accurate basis for perioperative nutritional intervention (Lau et al., 2017; Sabanayagam et al., 2020; Maheshwari et al., 2023). In the future, by constructing an interdisciplinary AI platform that integrates global stem cell research data, clinical cases, and patient information using natural language processing technology to standardize unstructured data; and mining potential associations through machine learning, this system will provide multi-dimensional data support for optimizing liver regeneration treatment strategies.

## 6 Discussion

The rapid development of stem cell research provides unprecedented opportunities for regenerative medicine and disease treatment; however, its clinical translation still faces multiple challenges. First, although iPSC technology has successfully avoided the ethical controversies of ESCs, its epigenetic instability and potential tumorigenicity remain the core issues restricting its widespread application (Sato et al., 2019). For example, residual pluripotency genes (such as c-Myc) during the reprogramming process may cause genomic abnormalities and lead to the formation of teratomas (Lemmens et al., 2023). Secondly, the insufficient differentiation efficiency and functional maturity of stem cells limit their therapeutic efficacy in tissue repair. Taking mesenchymal stem cells (MSCs) as an example, they are prone to differentiate into fibroblasts instead of functional hepatocytes in the liver injury microenvironment, further exacerbating fibrosis (Ichim et al., 2018), which indicates that the microenvironment regulation strategies need to be further optimized.

The integration of cutting-edge technologies provides new ideas for breaking through these bottlenecks. The combination of gene editing technology (such as CRISPR-Cas9) and iPSCs has successfully corrected mutations related to genetic diseases (Itoh et al., 2020). For example, the use of CRISPR/Cas9 technology to introduce heterozygous point mutations in the PRPF8 gene of normal induced pluripotent stem cell (iPSC) lines and establish PRPF8 gene mutant cell lines (CSUASOi012-A-2) has provided valuable cellular resources for studying the pathogenesis of retinitis pigmentosa (Chen et al., 2025). However, the off-target effects of gene editing still need to be strictly evaluated. Organoid technology studies the efficacy of human induced pluripotent stem cell-derived lung organoids (hiLOs) derived from NKX2.1+ lung progenitor cells and airway basal cells (hiBCs) as a 3D model by simulating the 3D structure of organ development. For instance, targeting the most common mutation, F508del, researchers have assessed CFTR modulator response through forskolin-induced swelling assay. ROC analysis of FIS assay results showed an optimal cutoff value of 1.21, a sensitivity of 65.9%, a specificity of 71.8%, and a prediction accuracy of 76.4% for the model. The results demonstrated that hiLOs can effectively model CF pathology and predict patient-specific responses to modulators (Demchenko et al., 2025), providing a more accurate model for disease mechanism research and drug screening (Hofbauer et al., 2021). Furthermore, 3D bioprinting technology can achieve personalized tissue construction (Omondi et al., 2024); however, these technical methods still have certain limitations. For example, although CRISPR-Cas9/iPSC can accurately correct genetic defects, they still face differences in gene correction efficiency and the possibility of off-target effects in different iPSC cell lines (Walsh and Jin, 2024). Although 3D bioprinting could facilitate personalized tissue manufacturing some material limitations persist. For instance, synthetic polymers implanted in the body could trigger immune responses and induce inflammation (Wang et al., 2024). Biomaterials have better biological activity, but cytotoxic products may be released during degradation, and challenges persist in large-scale production and consistent control of biomaterials (Yuan et al., 2024). Organoid models are able to generalize disease pathology but lack vascularization and immune components, thereby limiting translational relevance (Wen et al., 2024).

Future research should focus on interdisciplinary innovation. AI has great potential in stem cell research. For example, machine learning can predict differentiation pathways and optimize culture conditions by analyzing single-cell sequencing data (Sabanayagam et al., 2020). Additionally, combining biomaterials science with stem cell biology to develop bioengineered microenvironment scaffolds may enhance the directed differentiation and functional integration of stem cells. Furthermore, standardizing the stem cell preparation process and establishing a long-term safety evaluation system are the key to promoting clinical translation.

In conclusion, the success of stem cell research requires both in-depth exploration of basic mechanisms and precise application of technological advancements. Through interdisciplinary cooperation and technological innovation, it is expected to break through existing bottlenecks and achieve clinical translation of regenerative medicine.
